# A Conserved Role for SNX9-Family Members in the Regulation of Phagosome Maturation during Engulfment of Apoptotic Cells

**DOI:** 10.1371/journal.pone.0018325

**Published:** 2011-04-08

**Authors:** Johann Almendinger, Kimon Doukoumetzidis, Jason M. Kinchen, Andres Kaech, Kodi S. Ravichandran, Michael O. Hengartner

**Affiliations:** 1 Institute of Molecular Life Sciences, University of Zurich, Zurich, Switzerland; 2 Center for Cell Clearance and the Department of Microbiology, University of Virginia, Charlottesville, Virginia, United States of America; 3 Center for Microscopy and Image Analysis, University of Zurich, Zurich, Switzerland; 4 Molecular Life Sciences PhD Program, Life Science Zurich Graduate School, UZH/ETHZ, Zurich, Switzerland; University of Medicine and Dentistry of New Jersey, United States of America

## Abstract

Clearance of apoptotic cells is of key importance during development, tissue homeostasis and wound healing in multi-cellular animals. Genetic studies in the nematode *Caenorhabditis elegans* have identified a set of genes involved in the early steps of cell clearance, in particular the recognition and internalization of apoptotic cells. A pathway that orchestrates the maturation of phagosomes containing ingested apoptotic cells in the worm has recently been described. However, many steps in this pathway remain elusive. Here we show that the *C. elegans* SNX9-family member LST-4 (lateral signaling target) and its closest mammalian orthologue SNX33 play an evolutionary conserved role during apoptotic cell corpse clearance. In *lst-4* deficient worms, internalized apoptotic cells accumulated within non-acidified, DYN-1-positive but RAB-5-negative phagosomes. Genetically, we show that LST-4 functions at the same step as DYN-1 during corpse removal, upstream of the GTPase RAB-5. We further show that mammalian SNX33 rescue *C. elegans lst-4* mutants and that overexpression of truncated SNX33 fragments interfered with phagosome maturation in a mammalian cell system. Taken together, our genetic and cell biological analyses suggest that LST-4 is recruited through a combined activity of DYN-1 and VPS-34 to the early phagosome membrane, where it cooperates with DYN-1 to promote recruitment/retention of RAB-5 on the early phagosomal membrane during cell corpse clearance. The functional conservation between LST-4 and SNX33 indicate that these early steps of apoptotic phagosome maturation are likely conserved through evolution.

## Introduction

Apoptotic cell clearance is an important process during development, tissue homeostasis and wound healing [1], [2]. The nematode *C. elegans* is a useful in vivo model to study programmed cell death and the clearance of apoptotic cells, as large numbers of cells die during embryonic development and during oogenesis in the adult germ line [3]. During clearance of apoptotic cells, two partially redundant pathways comprised of CED-1/MEGF10/LRP1, CED-6/GULP and CED-7/ABCA1, and of MIG-2/RhoG, UNC-73/Trio, CED-2/CrkII, CED-5/DOCK180 and CED-12/ELMO respectively, regulate the small GTPase CED-10/Rac, which in turn directs the actin polymerization and membrane extension required to engulf the dying cell [4]. CED-10/Rac activity during corpse engulfment is also regulated by ABL-1/Abl and its target ABI-1/Abi [5], the RacGAP SRGP-1 [6] as well as through a non-canonical Wnt pathway [7].

Following internalization, the two small GTPases RAB-5 and RAB-7, together with many additional factors, sequentially control the maturation of the nascent phagosome, ultimately leading to lysosome fusion and corpse degradation [8]–[15] for review see add [16], [17]. How RAB-5 is recruited to early phagosomes is the subject of intensive study. We previously described an evolutionary conserved mechanism for the recruitment of RAB-5 to the early phagosome involving the large GTPase DYN-1/Dynamin and the phophatidylinositol-3 kinase VPS-34 [18], [10]. Zhou and colleagues recently extended these observations by demonstrating that the sorting nexin LST-4 also functions at this step, interacting physically with DYN-1 and promoting the fusion of the maturing phagosomes with endosomes and lysosomes [19].

In this study, we confirm these observations and extend our understanding of LST-4 function through a careful genetic and cell biological dissection of the early steps in phagosome maturation. We also show that a mammalian homologue of LST-4 not only can rescue the *C. elegans lst-4* mutant, but likely also is required for effective phagosome maturation in mammals. We thus propose that LST-4 constitutes another member of the evolutionarily conserved mechanism that drives early phagosome maturation following internalization of apoptotic cell corpses.

## Results

### *lst-4* controls maturation of apoptotic cell-containing phagosomes

**Figure 1:**
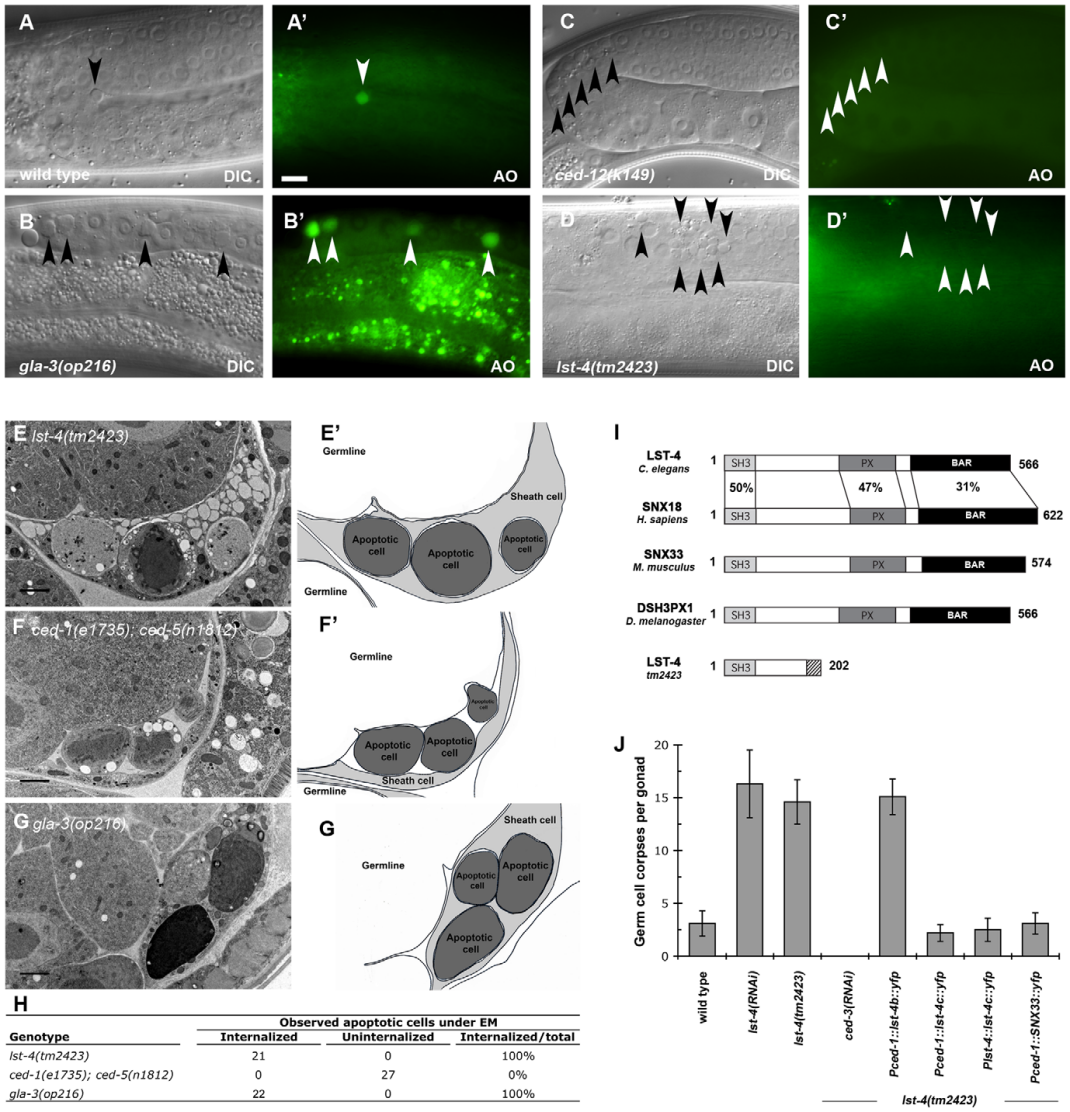
LST-4 is required for efficient cell corpse clearance in the adult *C. elegans* germ line. (**A**–**D**). DIC micrographs (**A**–**D**) or epifluorescence pictures (**A′**–**D′**) of *C. elegans* germlines. Dorsal is to the top and the germline bend is to right. Arrowheads indicate apoptotic germ cells or Acridine Orange (AO) staining of apoptotic corpses. In wild-type worms (**A**, **D′**) and in mutants with increased levels of germline apoptosis such as *gla-3(op216)* (**B**, **B′**), AO preferentially stains engulfed apoptotic cells present in acidic compartments. In worms mutant for genes required for efficient removal of apoptotic cells, here *ced-12(k149)*, refractile cell corpses persist but do not stain with AO (**C**, **C′**). Similarly, *lst-4(tm2423)* worms show increased persistent cell corpses that fail to stain with AO (**D**, **D′**). Size bar, 10 µm. (**E**–**G′**) Transmission electron microscopy images of cell corpses and their neighboring cells (**E**–**G**) and corresponding camera lucida drawings (**E**′–**G**′); apoptotic cells are represented in dark grey, sheath cells in light grey and the germline syncytium in white. In *lst-4(tm2423)* animals (**E**, **E**′), apoptotic cells are fully internalized by the sheath cells, whereas in the *ced-1(e1735)*; *ced-5(n1812)* double mutants apoptotic cells accumulate between germline syncytium and the sheath cells (**F**, **F**′). *gla-3(op216)* animals, which have increased germ cell apoptosis were used as a positive control (**G**, **G**′). Size bar, 2 µm. (**H**) Quantification of internalized apoptotic cells using TEM. For each genotype, two to three different animals 24 h post L4/adult molt were processed and analyzed as described by Zhou and coworkers [31]. (**I**) The domain structure of the SNX9 subfamily of sorting nexins is conserved through evolution. The percent aminoacid identity of each domain between LST-4 and human SNX18, mouse SNX33 and fly DSH3PX1 is indicated. SH3: Src- homology 3 domain; PX: Phagocytic oxidase domain; BAR: Bin/Amphiphysin/Rvs domain. The *tm2423* deletion results in a frame shift and a premature stop. (**J**) Expression of *C. elegans* LST-4 or mouse SNX33 in engulfing cells rescues the cell corpse clearance defect of *lst-4* mutants. Results shown are mean ± s.d. n>15 animals for each genotype.

DYN-1 contains a C-terminal proline-rich domain [20], which has been shown to bind to the SH3 domain of various signaling molecules [21]. To identify potential SH3 domain-containing binding partner(s) for DYN-1 in the phagosome maturation process, we conducted a targeted RNAi screen of *C. elegans* genes coding for SH3 domain-containing proteins (Fig. S1, Table S1). This candidate-based RNAi screen identified a single gene, *lst-4* (*l*ateral-*s*ignaling *t*arget) [22]. RNAi-mediated knockdown of *lst-4* led to a strong apoptotic cell corpse clearance defect (Fig. S1, Table S1). To confirm our RNAi results, we analyzed cell corpse clearance in animals carrying the *lst-4(tm2423)* mutation, a 212 bp deletion predicted to completely inactivate the gene (Fig. S2B). *lst-4(tm2423)* mutant worms showed a strong accumulation of non-acidified, AO-negative cell corpses in the hermaphrodite germline, a similar phenotype observed in *dyn-1(ky51)* mutants [10] (Fig. 1 A–D′, S1), suggesting a defect in cell corpse clearance upstream of phagosome acidification.

LST-4 belongs to the family of sorting nexins, a rather heterogeneous group of proteins unified by the presence of a particular type of phospholipid binding domain, the phox-homology (PX) domain [23]. We confirmed by RT-PCR and sequencing the existence of two of the four predicted LST-4 isoforms, LST-4b and LST-4c, which are generated through the use of alternative promoters (Fig. S2A). Both variants contain a PX and a BAR domain [24]; however only LST-4c also contains an additional N-terminal SH3 domain [25] (Fig. 1i, S2A, C). LST-4 is the only *C. elegans* homolog of the mammalian SNX9/SNX18/SNX33 subfamily of sorting nexins, referred to as the SNX9-family (Fig. 1i, S2C). In mammals, SNX9, SNX18 and SNX33 play various roles during endocytosis and endosomal sorting [26].

To address whether the persistent cell corpse phenotype observed in *lst-4(tm2423)* mutants is due to defects in corpse internalization or phagosome maturation, we quantified germ cell corpse internalization by transmission electron microscopy (Fig. 1E–H). Following apoptosis, dying germ cells are normally recognized, engulfed and gradually degraded by the surrounding somatic sheath cells [27] (Fig. 1G, G′, H). In *ced-1(e1735)*; *ced-5(n1812)* double mutant animals, where corpse internalization is blocked [4], apoptotic germ cells accumulate as unengulfed corpses between the germline syncytium and the gonadal sheath (Fig. 1F–F′, H). By contrast, in *lst-4(tm2423)* mutants, all apoptotic germ cells were internalized, but persisted as undegraded cell corpses within the sheath cells (Fig. 1E–E′, H). We also used fluorescent reporters to study the recruitment of the engulfment receptor CED-1 [9] and of F-actin around apoptotic cells [10]. Recruitment of these reporters was similar in *lst-4(tm2423)* mutants and wild-type animals (Fig. S3). Taken together, these data suggest that *lst-4* is not required for cell corpse recognition or internalization, but rather for the subsequent maturation of apoptotic cell-containing phagosomes.

### A conserved role of LST-4/SNX33 in phagosome maturation

We expressed splice variant LST-4c, which includes the N-terminal SH3 domain, as a C-terminal *yfp* fusion under the control of either the engulfment gene promoter P*_ced-1_* [28] or the endogenous promoter P*_lst-4_* (as described in materials and methods). Both constructs efficiently rescued the persistent cell corpse phenotype of *lst-4(tm2423)* mutant worms. Similar rescuing results were obtained by Lu *et al.* with isoform LST-4d [19], which differs from LST-4c by only two amino acids through the use of an alternative 5′-splice site for exon 5 (Fig. S2A). By contrast, isoform LST-4b, which lacks the SH3 domain, failed to rescue (as did LST-4c constructs lacking the SH3 domain, see below) (Fig. 1J). These results confirmed the importance of the SH3 domain of LST-4 and suggested that LST-4c and LST-4d are the most relevant isoforms for clearance of apoptotic corpses *in vivo*. Thus, we used isoform LST-4c for all subsequent experiments.

To date, all engulfment genes identified in the nematode context have also played an evolutionarily conserved role in corpse removal [29]–[31]. To address this issue, we expressed SNX33, the mouse SNX9-family member most similar to LST-4 (Fig. 1I), in the engulfing somatic sheath cells and observed a full rescue of *lst-4(tm2423)* mutants (Fig. 1J). This observation provides genetic evidence that the function of the SNX9-familiy member LST-4/SNX33 in cell corpse clearance is evolutionary conserved (see also below).

### LST-4/SNX33 co-localizes with DYN-1 on early phagosomes and is rapidly released upon RAB-5 recruitment

**Figure 2:**
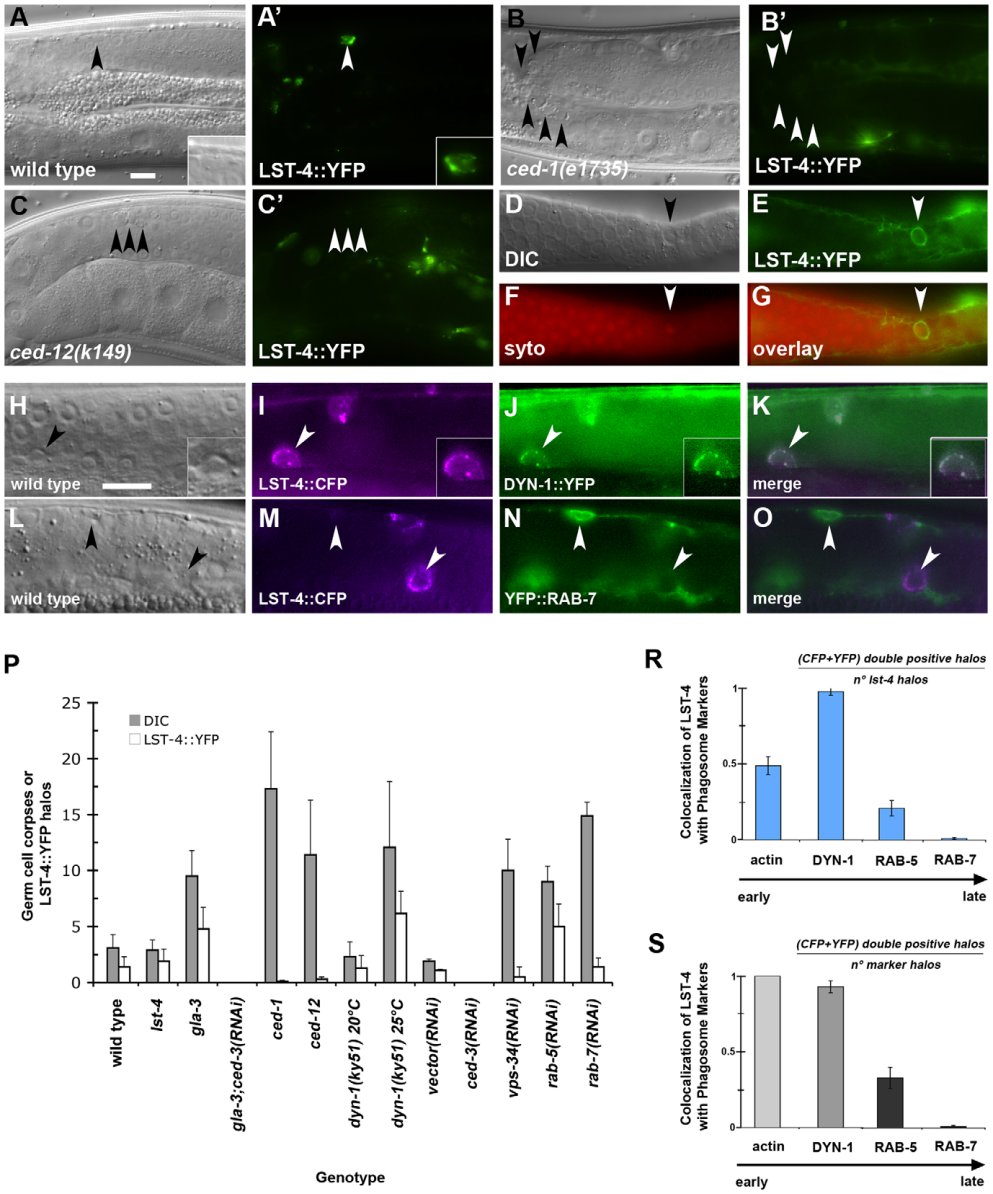
LST-4 is recruited to the early phagosome where it colocalizes with DYN-1 and RAB-5. (**A**–**G**) DIC micrographs (**A**–**D**) or epifluorescence pictures (**A′**–**C′**, **E**–**G**) of *C. elegans* germ lines. Arrowheads indicate early apoptotic germ cells or protein localized around apoptotic germ cells. LST-4 is recruited around the apoptotic cell during internalization (**A′**, **E**, **G** arrowhead) and highlights early, SYTO negative corpses (**F**). SYTO, like Acridine Orange, preferentially stains late-stage, internalized apoptotic cells. When the internalization process is disrupted, as in *ced-1(e1735)* (**B**, **B′**) and *ced-12(k149)* mutants (**C**, **C′**), LST-4 localization around apoptotic germ cells is lost (**B′**, **C′** arrowheads). (**H**–**O**) DIC micrographs (**H**, **L**) or epifluorescence pictures (**I**–**K**, **M**–**O**) of *C. elegans* germ lines. LST-4::CFP (**I**, **M**) extensively colocalizes with DYN-1::YFP (**J**, **K**), but not with YFP::RAB-7 (**N**, **O**). (**P**) Localization of LST-4::YFP in different genetic backgrounds. Germ cell corpses and LST-4 positive phagosomes were quantified as described in materials and methods. Data shown are mean ± s.d. n>15 animals for each condition. Size bars, 10 µm. (**R**, **S**) Colocalization index of LST-4::CFP with YFP::actin, YFP::DYN-1, YFP::RAB-5 and YFP::RAB-7. Data shown are mean ± s.e.m. n>50 halos for each genotype. Determination of the colocalization index is described in materials and methods.

To better understand at which stage of apoptotic cell processing/phagosome maturation LST-4 might play a role, we analyzed the subcellular distribution of LST-4c::YFP during the engulfment process. In our rescuing lines, we observed that LST-4 localized in a patchy pattern around early (non-condensed, SYTO-negative) apoptotic germ cells (Fig. 2D–G), similar to DYN-1 localization [10], [32]. These patches have been suggested to correspond to regions of extending tubules that attach and recruit surrounding endosomes and lysosomes [9], [19]. LST-4 was not recruited to apoptotic germ cells in *ced-1(e1735)* or *ced-12(k149)* mutant animals (Fig. 2A–C′), indicating that recruitment occurs downstream of corpse internalization. We also performed co-localization studies to increase the temporal resolution of LST-4 recruitment during phagosome maturation. LST-4 co-localized extensively with actin and DYN-1 around apoptotic cells (Fig. 2H–K, R, S) but only minimally with RAB-5 (Fig. 2R, S) and not at all with RAB-7 (Fig. 2L–O, R, S). Taken together, these results indicate that engulfment signaling is required for LST-4 recruitment and suggest that both LST-4 and DYN-1 act at an early step of phagosome maturation (Fig. 2I–K) [10].

We next tested the genetic requirements for LST-4 localization during phagosome maturation (Figure 2P). Previous studies have shown that DYN-1 associates with VPS-34 and that these two proteins together play a key role in the recruitment of RAB-5 to early phagosomes containing apoptotic corpses. Recruitment of LST-4c::YFP to phagosomes containing germ cell corpses was greatly diminished in *vps-34(RNAi)* animals, suggesting that VPS-34 is required for efficient recruitment or maintenance of LST-4 on early phagosome membranes. To determine whether DYN-1 function is required for LST-4 recruitment, we compared sub-cellular localization of LST-4c::YFP in *dyn-1(ky51ts)* animals at permissive and restrictive temperature. Whereas both germ cell corpse number and LST-4 localization were normal at the permissive temperature, we observed a large increase in LST-4-positive phagosomes at the restrictive temperature. A similar accumulation of LST-4-positive phagosomes was observed in *rab-5(RNAi)* animals. By contrast, RNAi knockdown of *rab-7*, which acts at a later step in the phagosome maturation pathway, led to animals with normal numbers of LST-4-positive phagosomes, despite the accumulation of a large number of (late stage) phagosomes. These results suggest that loss of *vps-34* arrests phagosomes at a very early step, prior to LST-4 recruitment, whereas inhibition of DYN-1 GTPase activity or loss of *rab-5* arrest at a later, LST-4-positive stage of phagosome maturation.

**Figure 3:**
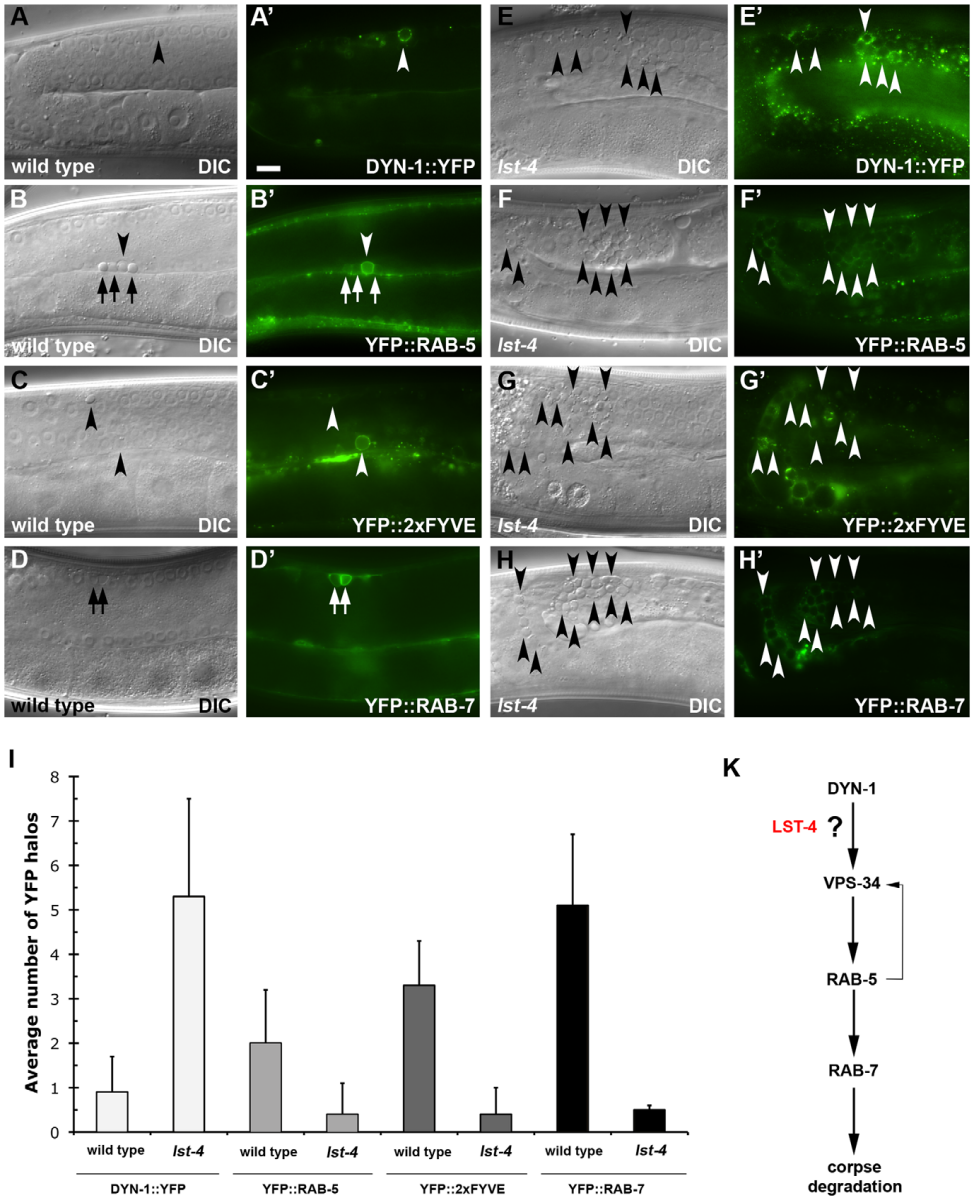
LST-4 function is required for DYN-1(+) to RAB-5(+) progression during phagosome maturation. (**A**–**H′**) DIC micrographs (**A**–**H**) or epifluorescence pictures (**A′**–**H′**) of wild type (**A**–**D′**) or *lst-4* mutant worms (**E**–**H′**) carrying different fluorescent reporters. Arrowheads indicate early apoptotic germ cells or protein localized around apoptotic germ cells. Arrows denote late apoptotic germ cells or fluorescent halos formed around them. In both wild type (**A′**) and *lst-4* mutants (**E′**) DYN-1::YFP is efficiently recruited to apoptotic cells whereas YFP::RAB-5 (**B′**, **F′**), YFP::2xFYVE (**C′**, **G′**) and YFP::RAB-7 (**D′**, **H′**) halos are less frequent in the gonad of *lst-4(tm2423)* mutant worms (**F′**, **G′**, **H′** arrowheads) than in wild type animals (**B′**, **C′**, **D′**). (**I**) LST-4 is required for efficient recruitment of RAB-5, 2xFYVE and RAB-7 to the phagosome as well as for the release, but not for the recruitment of DYN-1. Results shown are mean ± s.d. n>15 animals for each genotype. (**K**) Genetic pathway for phagosome maturation. LST-4 likely acts downstream or in parallel to DYN-1 and upstream of RAB-5. Size bar, 10 mm.

We also performed a complementary set of experiments to determine the requirement for LST-4 in the recruitment of various other fluorescent reporters to the maturing phagosome. In the *lst-4(tm2423)* mutants, decreased recruitment was observed for the small GTPases RAB-5 and RAB-7, which have been previously shown to localize to early and late phagosomes respectively, as well as for the 2xFYVE::YFP reporter, which monitors VPS-34 activity [10] (Fig. 3B–H′, I). By contrast, we observed an increase in CED-1 and in DYN-1-positive phagosomes in *lst-4* mutants, suggesting that DYN-1 recruitment to the phagosome membrane occurs independently of LST-4 function, and that in the absence of *lst-4* function, phagosomes lacking arrest at an early, CED-1-positive and DYN-1-positive, but RAB-5-negative stage of maturation (Fig. 3A–A′, E–E′, I). Taken together, these data indicate that LST-4 functions early in the phagosome maturation pathway, either together with or in parallel to DYN-1, but upstream of RAB-5, VPS-34 activity and RAB-7 (Fig. 3K). LST-4 is further required for the recruitment of RAB-5 to the maturing phagosome. Conversely, DYN-1 activity, as well as recruitment or activation of RAB-5 is required for removal of LST-4 from phagosomal membranes.

### LST-4/SNX33 PX and SH3 domains are required for function in corpse degradation

**Figure 4:**
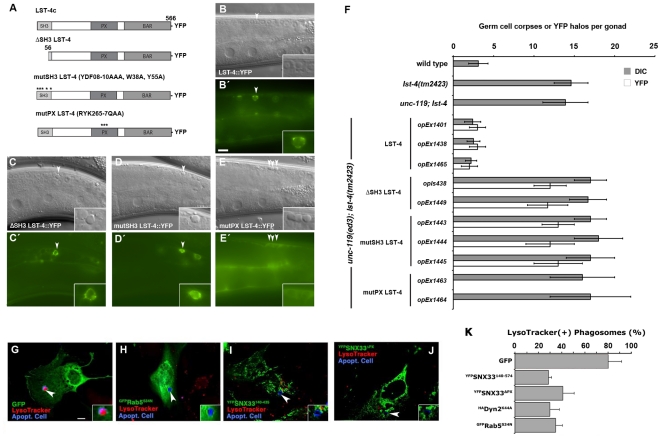
Structure function analysis of LST-4/SNX33. (**A**–**F**) LST-4c constructs (**A**) were tested for their ability to rescue *lst-4* mutants and for localization around internalized apoptotic cells (**A**–**D′**). DIC (**B**–**E**) and epifluorescence images (**B′**–**E′**) of transgenic lines expressing LST-4c::YFP (**B**, **B**′), ΔSH3 LST-4c::YFP (**C**, **C**′), mutSH3 LST-4c::YFP (**D**, **D**′) and mutPX LST-4c::YFP (**E**, **E**′). (**A**) Schematic of LST-4c rescuing constructs. (**F**) Quantification of germ cell corpses and LST-4c::YFP halos around apoptotic cells in the corresponding genetic backgrounds. Animals were scored 24 h post L4/adult molt under DIC and epifluorescence. Data shown are means ± SD, n>15 animals. Size bar, 10 µm. (**G**–**K**) The role of SNX33 in phagosome maturation is evolutionary conserved. NIH/3T3 fibroblasts transfected with GFP (**G**), ^GFP^Rab5^S34N^ (**H**), ^YFP^SNX33^140–435^ (**I**), ^YFP^SNX33^ΔPX^ (**J**), or ^HA^Dyn2^K44A^ were incubated with apoptotic thymocytes (shown in blue) and Lysotracker Red LTR to determine the efficiency of phagosome maturation. Cells transfected with ^YFP^SNX33^140–435^, ^YFP^SNX33^ΔPX^, ^GFP^Rab5^S34N^ and ^HA^Dyn2^K44A^ showed decreased numbers of engulfed thymocytes (arrows) co-staining with Lysotracker Red. Size bar, 10 µm. The percentage internalized apoptotic phagosomes that were also Lysotracker positive (i.e., matured into acidic phagolysosomes) are shown in (**K**) (mean ± s.d., n = 3 experiments with 60 cells scored; only phagosomes in transfected [GFP(+) or HA(+)] cells were scored).

We next performed a structure/function analysis of LST-4/SNX33 during cell corpse clearance. Expression of mutant versions of LST-4, such as LST-4^ΔSH3^, LST-4^mutSH3^ and LST-4^mutPX^ (bearing mutations in the SH3 or PX domain, Fig. 4A, 4E) could not rescue the *lst-4(tm2423)* persistent corpse phenotype (Fig. 4E–F). These results indicate that both the SH3 and PX domains of LST-4 are required for efficient phagosome maturation. Interestingly, we found that mutations in the SH3 domain did not affect LST-4 localization on the phagosome membrane (Fig. 4A–C′). In contrast, when we mutated the PX domain [33], LST-4 remained cytoplasmic and was no longer recruited to the phagosome. Thus, the putative interaction of DYN-1 with the LST-4 SH3 domain, while essential for LST-4 function, is not required for LST-4 recruitment to the phagosome membrane. Rather, the dependence on the PX domain suggests that LST-4 might be recruited directly to the early phagosome membrane through lipid binding. This result implies a mode of recruitment of LST-4 and DYN-1 during phagosome maturation that is distinct from the suggested mechanism of SNX9 recruitment during clathrin mediated endocytosis in mammals, where SNX9 and Dyn2 are recruited as a binary complex from the cytosol to the clathrin coat [26].

Since expression of mammalian SNX33 could rescue the phagosome maturation defect of *C. elegans lst-4(tm2423)* mutants, we surmised that SNX9-family members might also participate in mammalian apoptotic cell clearance. To test this hypothesis, we expressed various SNX33 constructs in NIH/3T3 fibroblasts and measured their ability to internalize and degrade apoptotic thymocytes. In contrast to control transfected NIH/3T3 cells, expression of ^YFP^SNX33^140–435^ lacking the SH3 domain or ^YFP^SNX33^ΔPX^ lacking the PX domain (Fig. 4G, I, J), significantly decreased the fraction of engulfed apoptotic thymocytes that were present within late (acidified) phagosomes (Fig. 4 G, K). Similar results were obtained through expression of the dominant negative constructs ^GFP^Rab5^S34N^ or Dyn2^K44A^ (Fig. 4H, K). These results suggest that expression of truncated versions of SNX33 inhibits, directly or indirectly, the maturation of phagosomes containing internalized apoptotic cells.

## Discussion

In this paper, we used a combination of genetic and cell biological studies in *C. elegans* to better define the function of the sorting nexin LST-4 in apoptotic cell corpse clearance. We also show, using transgenic rescue and mammalian cell culture experiments that the role of the LST-4/SNX-33 sorting nexin family in the maturation of apoptotic cell-containing phagosomes in conserved through evolution.

Our data suggest a model in which LST-4 and DYN-1 are among the first proteins to be recruited to the nascent phagosome. Is it possible to order these two proteins? Our observation of the accumulation of DYN-1-positive phagosomes in the adult gonad of *lst-4(tm2423)* clearly indicate that DYN-1 can be recruited to phagosomes in the absence of *lst-4* function (Figure 3). Lu *et al.* also observed recruitment of DYN-1 to early phagosomes during embryonic development, albeit it at a reduced efficiency [19]. These observations suggest that DYN-1 is the first currently known protein to be recruited to the nascent phagosome.

Recruitment of LST-4 to phagosomes appears to be more complex. Although DYN-1 and LST-4 are known to interact physically in both *C. elegans* and in mammals [19], [34], we found that recruitment of LST-4 to phagosomes *in vivo* was dependent on the phosphoinositide-binding PX domain but not on the SH3 domain known to mediate interaction with DYN-1 (Figure 4). Interaction with DYN-1 (possibly via another interaction domain) might still be important however, as Lu and colleagues reported a complete lack of LST-4 recruitment in *dyn-1(en9)* embryos, in which DYN-1 fails to associate with phagosomes [19]. Surprisingly, we observed the opposite phenotype, namely an accumulation of LST-4-positive phagosomes in the gonad of animals carrying the *dyn-1(ky51ts)* mutation. This apparent contradiction can likely be resolved thanks to the recent discovery from the Zhou group that *dyn-1* mutants that fail to self-assemble (e.g., *en9*) have different set of phagosome maturation defects than those that are defective in GTP hydrolysis (such as *ky51*): whereas the former mutants fail to associate with phagosomes and prevent efficient recruitment of RAB-7, the latter mutants can bind to phagosomes, but fail to dissociate [13].

Based on these combined data, we propose that the physical presence of DYN-1 on phagosomes likely is essential for recruitment of LST-4, either through a direct interaction via a domain other than the SH3 domain, or through activation of other factors required for LST-4 recruitment, such as VPS-34 (Figure 2, 4). Importantly, although the LST-4 SH3 domain is not required for recruitment of LST-4 to phagosomes, it is essential for LST-4 function, since LST-4 protein lacking the SH3 domain fails to rescue *lst-4(tm2423)* mutants.

Both our kinetic analysis of phagosome maturation in the adult gonad (Figure 2, [10]) and the time course studies by Zhou and colleagues [13], [19], [32] show that DYN-1 and LST-4 only transiently associate with the maturing phagosome. We show above that dissociation of DYN-1 is dependent on LST-4 (Figure 3) and that conversely, dissociation of LST-4 requires a GTPase-competent DYN-1 (Figure 2). A similar relationship holds between LST-4, DYN-1, and the GTPase RAB-5: not only are both *lst-4* and *dyn-1* required for efficient recruitment to phagosomes (Figure 2, [10], [19]), but both LST-4 and DYN-1 also fail to dissociate in the absence of RAB-5 (Figure 2, [10]). These observations suggest that LST-4 and DYN-1 coordinate, likely as part of a complex (Figure 4; [19]) RAB-5 recruitment. Once present on the phagosome, RAB-5 could then in turn, directly or indirectly, promote dissociation of DYN-1 and LST-4, possibly through promotion of DYN-1 GTPase activity [13].

**Figure 5:**
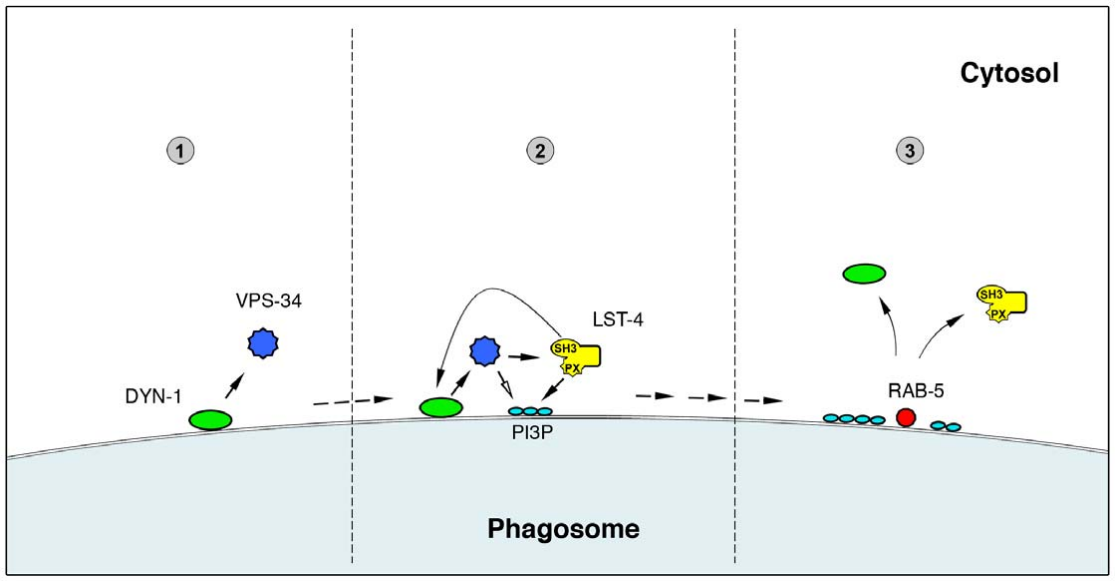
Model of early phagosome maturation. 1. DYN-1 is recruited to nascent phagosomes and subsequently recruits VPS-34. 2a. VPS-34 in turn promotes the recruitment of LST-4 through direct interaction and/or the production of phasphatidylinositol-3 phosphates (open arrow) to which LST-4 binds via its PX domain. 2b. Membrane bound LST-4 associates physically with DYN-1 to generate an active complex that promotes RAB-5 recruitment and further phagosome maturation. 3. The presence of active RAB-5 on the phagosome membrane stimulates the inactivation and release of the DYN-1/LST-4 complex from mature phagosomes.

The results presented here, together with previous data from our and other groups, suggest a sequential recruitment model for early phagosome maturation, where DYN-1 binds to and recruits VPS-34, which then through direct interaction and/or phosphoinositide-3-phosphate [PtdIns(3)P] production recruits LST-4 to the phagosome. Once on the phagosome surface, LST-4 interacts via its SH3 domain with DYN-1, generating an activated complex able to promote further maturation steps, including recruitment of RAB-5 and RAB-7. Finally, RAB-5 recruitment to and/or its activation on the early phagosome membrane likely promotes both the release of LST-4 and DYN-1 and progression through further downstream maturation events (Figure 5).

Interestingly, the degradation of apoptotic cells utilizes many genes that also function in receptor-mediated endocytosis, and their mutation can result in various human diseases (e.g., mammalian orthologues of RAB-5 in tuberous sclerosis, Dynamin-2 and Rab7 in Charcot-Marie-Tooth disease [35]–[37]). Additionally, defects in phagocytic processes related to corpse removal result of autoimmune diseases and impaired neuronal function [31], [38], [39] and several players involved in this process have been implicated in pathogenesis of infectious agents [40]–[42]. Thus a better understanding of the molecular basis of apoptotic cell degradation could be of key importance to multiple aspects of normal cellular homeostasis and disease.

## Methods

### Nematode strains and reagents

Nematode strains were cultivated as described previously [27]. Mutations used were as follows: LGI: *gla-3(op216)*, *ced-1(e1735)*; LGIII: *ced-6(n1813)*, *opIs110 [Plim-7::yfp::act-5]*; LG IV: *lst-4(tm2423)*, LGV: *bcIs39 [Plim-7::ced-1::gfp]*, LGX: *dyn-1(ky51ts)*. The *ky51* allele is a temperature-sensitive mutation in the GTPase domain of DYN-1, resulting in a rapid and reversible functional inactivation of the protein at the restrictive temperature [20]. Integration sites of *opIs220 [P_eft-3_::dyn-1::yfp, unc-119(+)]*, *opIs223 [P_eft-3_::yfp::rab-7, unc-119(+)]*, *opIs282 [P_ced-1_::yfp::rab-5, unc-119(+)]*, *opIs436 [P_ced-1_::mSNX33::yfp, unc-119(+) – line1]*, *opIs437 [P_ced-1_::mSNX33::yfp, unc-119(+) – line2]* and *opIs438 [P_ced-1_::lst-4c_ΔSH3::yfp, unc-119(+)]* were not mapped. Unless noted otherwise, mutations were described previously [43]. *opEx1401 [P_ced-1_::lst-4c::yfp, unc-119(+)]*, *opEx1402 [P_ced-1_::lst-4b::yfp, unc-119(+)]*, *opEx1438 [P_lst-4_::lst-4c::yfp, unc-119(+) – line1]*, *opEx1465 [P_lst-4_::lst-4c::yfp, unc-119(+) – line2]*, *opEx1439 [P_ced-1_::lst-4c::cfp, unc-119(+)]*, *opEx1443 [P_ced-1_::lst-4c_mutSH3::yfp, unc-119(+) – line1]*, *opEx1444 [P_ced-1_::lst-4c_mutSH3::yfp, unc-119(+) – line2]*, *opEx1445 [P_ced-1_::lst-4c_mutSH3::yfp, unc-119(+) – line3]*, *opEx1463 [P_lst-4_::lst-4c_mutPX::yfp, unc-119(+) – line1]*, *opEx1464 [P_lst-4_::lst-4c_mutPX::yfp, unc-119(+) – line2]*, are extrachromosomal arrays.

### Reverse genetic screen

Feeding RNAi was performed as previously described [10]. Plates containing NGM-agarose, 200 mg/ml Ampicillin (Amp) and 2 mM IPTG (RNAi plates) were inoculated with 300 ml of appropriate bacterial cultures (transformed with constructs for generation of double stranded RNA under the control of the T7 promoter) and incubated for 8–12 hours before addition of worms. Between 30–60 synchronized *gla-3(op216)* L1-stage worms (*gla-3* mutants have an increased number of apoptotic germ cells [44] were placed on each RNAi plate and left for 72 h at 20°C [45]. Worms were then stained with acridine orange and apoptotic germ cells scored under an M2Bio Epifluorescence dissecting microscope (Zeiss). Positive candidates were retested and apoptotic germ cells scored directly by DIC microscopy.

### DIC and immunofluorescence microscopy

Worms were placed on 2% agarose pads in M9, anaesthetized with levamisole (3–5 mM; Sigma) and mounted under a coverslip for observation using a Leica DM-RA microscope equipped with DIC (Normarski) optics and standard epifluorescence with filters appropriate for detection of YFP, CFP/SYTO41, SYTO59 or GFP. Images were false colored in OpenLab or Adobe Photoshop 11.0.1, which was also used to optimize brightness and contrast. Acridine orange (Molecular probes) staining was performed as described previously [46]. To observe engulfed cells, gonads were dissected in PBS supplemented with 12.5 µM of SYTO41 or SYTO59 (Invitrogen), incubated in the dark for 10 min and then scored under a fluorescence microscope.

### Transmission electron microscopy

*C. elegans* grown on *E. coli* on NGM agar plates were high-pressure frozen with an EM Pact2 (Leica Microsystems, Vienna, Austria) using flat specimen carriers with an indentation of 1.5 mm×0.2 mm. The hole of the carrier (dedicated for pressure transmission) was filled with 1-hexadecene and the cavity of the carrier with a droplet of PBS. *C. elegans* were picked from the agar plate and transferred to the PBS droplet. Subsequently, the majority of the buffer solution was drawn off with a filter paper leaving the worms in a small volume of buffer. 1-hexadecene was added on top and the specimen was frozen immediately. The frozen specimens were freeze-substituted in anhydrous acetone containing 2% OsO_4_ in a Leica EM AFS2 freeze-substitution unit (Leica Microsystems). Specimens were kept successively at −90°C, −60°C, and at −30°C for 8 hours each. Temperatures were changed at a rate of 30°C per hour, finally reaching room temperature. After keeping the specimens at room temperature for 1 hour, OsO_4_ was removed by washing the specimens with anhydrous acetone twice. Subsequently, the specimens were gradually embedded in Epon/Araldite (Sigma-Aldrich, Buchs, Switzerland), 33% in anhydrous acetone overnight at 4°C, 66% in anhydrous acetone for 6 hours at 4°C and 100% for one hour at room temperature prior to polymerization at 60°C for 48 hours. Thin sections were stained with aqueous uranyl acetate 2% and Reynolds lead citrate and imaged in a Philips CM 100 transmission electron microscope (FEI, Eindhoven, Netherlands) using a Gatan Orius CCD camera and digital micrograph acquisition software (Gatan GmbH, Munich, Germany).

### Generation of transgenic nematodes

Transgenic worms were obtained by micro-particle bombardment in a Biolistic PDS-1000 (Bio-Rad) transformator as extrachromosomal arrays (*opEx*) or low-copy chromosomal integrants (*opIs* alleles). *unc-119* was used as a transformation marker. All lines were subsequently tested for rescue in the *unc-119(ed3)*; *lst-4 (tm2423)* mutant background (see Results).

### Plasmid construction

See additionally Table S1. Predicted isoforms of *lst-4* (*b* and *c*) were amplified by RT-PCR from an N2 *cDNA* library using primers that added an AscI site upstream and an FseI site downstream of the coding sequence. PCR products were subcloned into pJET1/blunt or pJET1.2/blunt (Fermentas) and sequenced. The resulting plasmids (pJA17–18) were digested by AscI and FseI (New England Biolabs) and cloned into the bombardment vector: pLN180 (which contains *ced-1* promoter, a C-terminal *yfp* cassette and the 3′ untranslated region of *let-858*) and pLN178 (as above, but with a c-terminal *cfp* cassette) to generate plasmid pJA20–21 and pJA36. *SNX33* open reading frame was amplified from pcDNA5/FRT/TOFLAG-mLST4 using the same strategy, to generate pJA42. A 4 kb P*_lst-4_* promoter region and 847 bp *lst-4* 3′UTR and downstream region were amplified from genomic N2 wild type DNA using primers which added for P*_lst-4_* a SbfI and an AscI cassette up- and downstream and SpeI and ApaI cassettes for *lst-4* 3′UTR respectively. The PCR products were cloned to generate pJA25 and pJA78. The SH3 deletion construct was performed using the forward primer *lst-4*_dSH3_AscI using pJA17 as a template, followed by cloning into pLN180 to generate pJA47. To generate mutations YDF8-10AAA, W38A and Y55A in the SH3 domain of LST-4c, DpnI-mediated site directed mutagenesis was performed starting from pJA17 using the oligos SDM_SH3_*lst-4*_1, 2 and 3. For the RYK265-7QAA mutation in the PX domain of LST-4c, ligation PCR was performed using the oligos SDM6b_PX*lst-4*_fw and SDM6bPX*lst-4*_rv. The PCR products were subcloned into pJET1.2, confirmed and further cloned into either pJA20 or pJA78 to generate pJA54 and pJA88, respectively.

Mouse SNX33 cDNA was purchased from Open Biosystems; the full-length cDNA was amplified by PCR and cloned into pcDNA5-FLAG or pEBB-HA as a KpnI – NotI fragment. C-terminal truncation (SNX33^140–574^) and SNX33^ΔPX^ constructs were generated by PCR with appropriate primers. The N-terminal expression construct containing the SH3 domain (SNX33^1–90^) was created using an ApaI site to remove residues 91–574. pcDNA5-FLAG-Vps34 is described previously [47].

### Lysotracker acidification assay

Cells were transfected using Lipofectamine 2000 as described previously [10], then washed and incubated in DMEM containing 10% fetal bovine serum (FBS) for approximately 8 h before the engulfment assay was conducted. Apoptotic thymocytes and Jurkat cells were generated as described previously [48]; apoptotic thymocytes (5.0×10^5^ cells per condition) were stained with either CellTracker Violet (Invitrogen) and added to NIH/3T3 cells in 4-well Labtek II culture chambers (Fisher) followed by a brief centrifugation to pellet cells onto the slide. Thymocytes were allowed to be engulfed for 30 min; unbound apoptotic thymocytes were gently washed off with DMEM containing 10% FBS; slides were then incubated for 2 h in the presence of Lysotracker Red (1∶10,000 dilution). Cells were then fixed with 3% paraformaldehyde (Sigma) in PBS for 30 min, permeabilized with 0.1% Triton X-100 (Sigma) and blocked with 5% skimmed milk that had been clarified by high speed centrifugation. Antibody staining was then performed as described previously [49]. Images were acquired using a Zeiss AxioImager microscope with standard filtersets for YFP (Alexa 488 anti-GFP or Alexa 488 anti-HA, Invitrogen), CFP (CellTracker Violet) and Cy3 (LysoTracker Red), then deconvolved to remove out-of-plane light in AxioVision (Zeiss AG).

## Supporting Information

Figure S1 **Identification of LST-4 from a targeted RNAi screen in** ***C. elegans*** **.** (**A**) Schematic of the candidate based reverse genetic screen. The *C. elegans* DYN-1 contains beside other domains, a C-terminal proline rich domain (PRD) [1]. PRDs are known to bind to SH3 domains of effector proteins [2]. We thus set up a list of all *C. elegans* genes encoding for SH3 domain-containing proteins in order to identify effector proteins that might function together with DYN-1 during cell corpse clearance. As previously described *dyn-1* (RNAi) treated or *dyn-1(ky51)* mutant worms at the non-permissive temperature accumulate engulfed apoptotic germ cell corpses within non-acidified (Acridine Orange negative) phagosomes [3]. In a first round of screening we thus fed bacteria expressing dsRNA using the Ahringer RNAi feeding library [4] for our candidate genes to *gla-3(op216)* mutant worms at the L1 larval stage, then adults were stained with acridine orange (AO). *gla-3(op216)* mutant worms were used to increase the number of apoptotic cell corpses to allow the observation of acridine orange staining under a dissecting microscope. Candidates, which were able to suppress AO staining, underwent a second round of screening in N2 wild type animals and analyzed for increased germ cell corpses in the adult hermaphrodite germ line. (**B**) DIC micrographs of *C. elegans* adult hermaphrodites germ lines at the stage 24 h post L4/adult molt. RNAi was performed on N2 wild type animals as previously described. Compared to control RNAi, RNAi-mediated knockdown of *lst-4* resulted in persistent cell corpses, a phenotype which is similar to *dyn-1(ky51)* mutants at the non-permissive temperature. Interestingly in *lst-4(RNAi)* or *lst-4(tm2423)* animals we were unable to observe any obvious defect in the clearance of embryonic cell corpses. It might be that *lst-4* is not expressed, or perhaps simply redundant under these conditions. The latter hypothesis is supported by recent work from Yang and coworkers, who found that the persistent cell corpse defect of retromer mutants was enhanced upon loss of *lst-4* function [5]. Scale bar, 10 mm. (For Supplemental References see File S1).
(TIF)

Figure S2 **Alignment and molecular nature of** ***lst-4*** **and its allele** ***tm2423*** **.** (**A**) The *C. elegans lst-4* locus is predicted to code for at least four different isoforms: *lst-4a*, *lst-4b*, *lst-4c* and *lst-4d*. We confirmed by RT-PCR amplification and subsequent sequencing the exon/intron structure for the two isoforms *lst-4b* and *lst-4c*. Boxes represent exons; the regions coding for the SH3, the PX or the BAR domain are highlighted in grey, white boxes represent 3′ untranslated regions. Thin lines represent introns. *tm2423* is a 212 bp deletion that results in a frame shift and premature termination (red bar). The positions of the primers used for genotyping *tm2423* are indicated. (**B**) Genotyping of *lst-4(tm2423)* and wild type animals by PCR amplification. Primer sequences are described in Table S2. (**C**) Protein sequence alignment of *C. elegans* LST-4c with human SNX18, mouse SNX33, and *Drosophila* DSH3PX1. All proteins contain a similar protein architecture consisting of a conserved N-terminal SH3 domain, a middle PX domain and a C-terminal Bar domain (indicated by thin lines). The thick line indicates the location of the *tm2423* deletion. The *tm2423* deletion results in truncated protein lacking the PX domain and the whole C-terminal part (Fig. 1I).
(TIF)

Figure S3 **Corpses are efficiently recognized and internalized in** ***lst-4(tm2423)*** **mutant worms.** (**A**–**D**) DIC (**A′**–**D′**) and epifluorescence images of CED-1::GFP (**A′**, **B′**) and YFP::actin (**C′**, **D′**) in wild-type (**A**, **C**) and *lst-4* mutants (**B**, **D**). Arrowheads indicate apoptotic germ cells or protein around apoptotic germ cell. In *lst-4(tm2423)* mutants, the recruitment of CED-1::GFP (**B′**) and the reorganization of YFP::actin (**D′**) during engulfment appear normal. Size bar, 10 mm. (**E**, **F**) Quantification of germ cell corpses and CED-1::GFP (**E**) or YFP::actin halos (**F**) around apoptotic cells in the indicated genetic backgrounds. Animals were scored 24 h post L4/adult molt under DIC and epifluorescence. Data shown are means ± SD, n>15 animals.
(TIF)

Table S1 **List of** ***C. elegans*** **genes, encoding SH3-containing proteins, screened for suppression of AO staining.** RNAi was performed in the *gla-3(op216)* background (to asses suppression of AO) or in wild-type worms where applicable (to quantify persistent cell corpses) as described in materials and methods. Only RNAi against *lst-4* was found to potently suppress AO staining of apoptotic germ cell corpses and to provoke a strong cell corpse accumulation in the *C. elegans* germline. The known SH3 domain containing engulfment genes *ced-2* and *ced-5* were not identified in this screen, likely due to the variable penetrance of feeding RNAi against these two genes. Clone source: Ahringer: plasmids from the Ahringer RNAi library [6]. pKD clones: genomic fragments from the gene of interest were PCR amplified and cloned into the RNAi feeding vector L4440. n.d., not done. (For Supplemental References see File S1).
(TIF)

Table S2 **List of Primers and Plasmids used in this study.**
(TIF)

File S1 **Supplemental References.**
(DOCX)

## References

[1] Henson PM, Hume DA (2006). Apoptotic cell removal in development and tissue homeostasis.. Trends Immunol.

[2] Ravichandran KS, Lorenz U (2007). Engulfment of apoptotic cells: signals for a good meal.. Nat Rev Immunol.

[3] Lettre G, Hengartner MO (2006). Developmental apoptosis in C. elegans: a complex CEDnario.. Nat Rev Mol Cell Biol.

[4] Kinchen JM, Cabello J, Klingele D, Wong K, Feichtinger R (2005). Two pathways converge at CED-10 to mediate actin rearrangement and corpse removal in C. elegans.. Nature.

[5] Hurwitz ME, Vanderzalm PJ, Bloom L, Goldman J, Garriga G (2009). Abl kinase inhibits the engulfment of apoptotic [corrected] cells in Caenorhabditis elegans.. PLoS Biol.

[6] Neukomm LJ, Frei AP, Cabello J, Kinchen JM, Zaidel-Bar R (2011). Loss of the RhoGAP SRGP-1 promotes the clearance of dead and injured cells in Caenorhabditis elegans.. Nat Cell Biol.

[7] Cabello J, Neukomm LJ, Günesdogan U, Burkart K, Charette SJ (2010). The Wnt Pathway Controls Cell Death Engulfment, Spindle Orientation, and Migration through CED-10/Rac.. PLoS Biol.

[8] Mangahas PM, Yu X, Miller KG, Zhou Z (2008). The small GTPase Rab2 functions in the removal of apoptotic cells in Caenorhabditis elegans.. J Cell Biol.

[9] Yu X, Lu N, Zhou Z (2008). Phagocytic receptor CED-1 initiates a signaling pathway for degrading engulfed apoptotic cells.. PLoS Biol.

[10] Kinchen JM, Doukoumetzidis K, Almendinger J, Stergiou L, Tosello-Trampont A (2008). A pathway for phagosome maturation during engulfment of apoptotic cells.. Nat Cell Biol.

[11] Lu Q, Zhang Y, Hu T, Guo P, Li W (2008). C. elegans Rab GTPase 2 is required for the degradation of apoptotic cells.. Development.

[12] Li W, Zou W, Zhao D, Yan J, Zhu Z (2009). C. elegans Rab GTPase activating protein TBC-2 promotes cell corpse degradation by regulating the small GTPase RAB-5.. Development.

[13] He B, Yu X, Margolis M, Liu X, Leng X (2010). Live-cell imaging in Caenorhabditis elegans reveals the distinct roles of dynamin self-assembly and guanosine triphosphate hydrolysis in the removal of apoptotic cells.. Mol Biol Cell.

[14] Kinchen JM, Ravichandran KS (2010). Identification of two evolutionarily conserved genes regulating processing of engulfed apoptotic cells.. http://www.ncbi.nlm.nih.gov/pubmed/20305638.

[15] Nieto C, Almendinger J, Gysi S, Gómez-Orte E, Kaech A (2010). ccz-1 mediates the digestion of apoptotic corpses in C. elegans.. J Cell Sci.

[16] Zhou Z, Yu X (2008). Phagosome maturation during the removal of apoptotic cells: receptors lead the way.. Trends Cell Biol.

[17] Kinchen JM (2010). A model to die for: signaling to apoptotic cell removal in worm, fly and mouse.. http://www.ncbi.nlm.nih.gov/pubmed/20461556.

[18] Huynh KK, Grinstein S (2008). Phagocytosis: dynamin's dual role in phagosome biogenesis.. Curr Biol.

[19] Lu N, Shen Q, Mahoney TR, Liu X, Zhou Z (2010). Three Sorting Nexins Drive the Degradation of Apoptotic Cells in Response to PtdIns(3)P Signaling.. Mol Biol Cell.

[20] Clark SG, Shurland DL, Meyerowitz EM, Bargmann CI, van der Bliek AM (1997). A dynamin GTPase mutation causes a rapid and reversible temperature-inducible locomotion defect in C. elegans.. Proc Natl Acad Sci USA.

[21] Gout I, Dhand R, Hiles ID, Fry MJ, Panayotou G (1993). The GTPase dynamin binds to and is activated by a subset of SH3 domains.. Cell.

[22] Yoo AS, Bais C, Greenwald I (2004). Crosstalk between the EGFR and LIN-12/Notch pathways in C. elegans vulval development.. Science.

[23] Lemmon MA (2008). Membrane recognition by phospholipid-binding domains.. Nat Rev Mol Cell Biol.

[24] Pylypenko O, Lundmark R, Rasmuson E, Carlsson SR, Rak A (2007). The PX-BAR membrane-remodeling unit of sorting nexin 9.. EMBO J.

[25] Musacchio A, Noble M, Pauptit R, Wierenga R, Saraste M (1992). Crystal structure of a Src-homology 3 (SH3) domain.. Nature.

[26] Lundmark R, Carlsson SR (2009). SNX9 - a prelude to vesicle release.. J Cell Sci.

[27] Gumienny TL, Lambie E, Hartwieg E, Horvitz HR, Hengartner MO (1999). Genetic control of programmed cell death in the Caenorhabditis elegans hermaphrodite germline.. Development.

[28] Zhou Z, Hartwieg E, Horvitz HR (2001). CED-1 is a transmembrane receptor that mediates cell corpse engulfment in C. elegans.. Cell.

[29] Metzstein MM, Stanfield GM, Horvitz HR (1998). Genetics of programmed cell death in C. elegans: past, present and future.. Trends Genet.

[30] He B, Lu N, Zhou Z (2009). Cellular and nuclear degradation during apoptosis.. Curr Opin Cell Biol.

[31] Nagata S, Hanayama R, Kawane K (2010). Autoimmunity and the clearance of dead cells.. Cell.

[32] Yu X, Odera S, Chuang C, Lu N, Zhou Z (2006). C. elegans Dynamin Mediates the Signaling of Phagocytic Receptor CED-1 for the Engulfment and Degradation of Apoptotic Cells.. Developmental Cell.

[33] Yarar D, Surka MC, Leonard MC, Schmid SL (2008). SNX9 activities are regulated by multiple phosphoinositides through both PX and BAR domains.. Traffic.

[34] Håberg K, Lundmark R, Carlsson SR (2008). SNX18 is an SNX9 paralog that acts as a membrane tubulator in AP-1-positive endosomal trafficking.. J Cell Sci.

[35] Houlden H, King RHM, Muddle JR, Warner TT, Reilly MM (2004). A novel RAB7 mutation associated with ulcero-mutilating neuropathy.. Ann Neurol.

[36] Meggouh F, Bienfait H, Weterman MA, de Visser M, Baas F (2006). Charcot-Marie-Tooth disease due to a de novo mutation of the RAB7 gene.. Neurology.

[37] Verhoeven K, De Jonghe P, Coen K, Verpoorten N, Auer-Grumbach M (2003). Mutations in the small GTP-ase late endosomal protein RAB7 cause Charcot-Marie-Tooth type 2B neuropathy.. Am J Hum Genet.

[38] Kawane K, Ohtani M, Miwa K, Kizawa T, Kanbara Y (2006). Chronic polyarthritis caused by mammalian DNA that escapes from degradation in macrophages.. Nature.

[39] Fuentes-Medel Y, Logan MA, Ashley J, Ataman B, Budnik V (2009). Glia and muscle sculpt neuromuscular arbors by engulfing destabilized synaptic boutons and shed presynaptic debris.. PLoS Biol.

[40] Mallo GV, Espina M, Smith AC, Terebiznik MR, Alemán A (2008). SopB promotes phosphatidylinositol 3-phosphate formation on Salmonella vacuoles by recruiting Rab5 and Vps34.. J Cell Biol.

[41] Flannagan RS, Cosio G, Grinstein S (2009). Antimicrobial mechanisms of phagocytes and bacterial evasion strategies.. Nat Rev Micro.

[42] Mercer J, Schelhaas M, Helenius A (2010). Virus entry by endocytosis.. Annu Rev Biochem.

[43] Bieri T, Blasiar D, Ozersky P, Antoshechkin I, Bastiani C (2007). WormBase: new content and better access.. Nucleic Acids Res.

[44] Kritikou EA, Milstein S, Vidalain P, Lettre G, Bogan E (2006). C. elegans GLA-3 is a novel component of the MAP kinase MPK-1 signaling pathway required for germ cell survival.. Genes Dev.

[45] Brenner S (1974). The genetics of Caenorhabditis elegans.. Genetics.

[46] Lettre G, Kritikou EA, Jaeggi M, Calixto A, Fraser AG (2004). Genome-wide RNAi identifies p53-dependent and -independent regulators of germ cell apoptosis in C. elegans.. Cell Death Differ.

[47] Peplowska K, Cabrera M, Ungermann C (2008). UVRAG reveals its second nature.. Nat Cell Biol.

[48] Park D, Tosello-Trampont A, Elliott MR, Lu M, Haney LB (2007). BAI1 is an engulfment receptor for apoptotic cells upstream of the ELMO/Dock180/Rac module.. Nature.

[49] Tosello-Trampont A, Kinchen JM, Brugnera E, Haney LB, Hengartner MO (2007). Identification of two signaling submodules within the CrkII/ELMO/Dock180 pathway regulating engulfment of apoptotic cells.. Cell Death Differ.

