# Supplementary figures and images for: A Conserved Role for SNX9-Family Members in the Regulation of Phagosome Maturation during Engulfment of Apoptotic Cells
Source: PLoS One. 2011 Apr 8;6(4):e18325. doi: 10.1371/journal.pone.0018325 (PMC3072968; doi:10.1371/journal.pone.0018325)

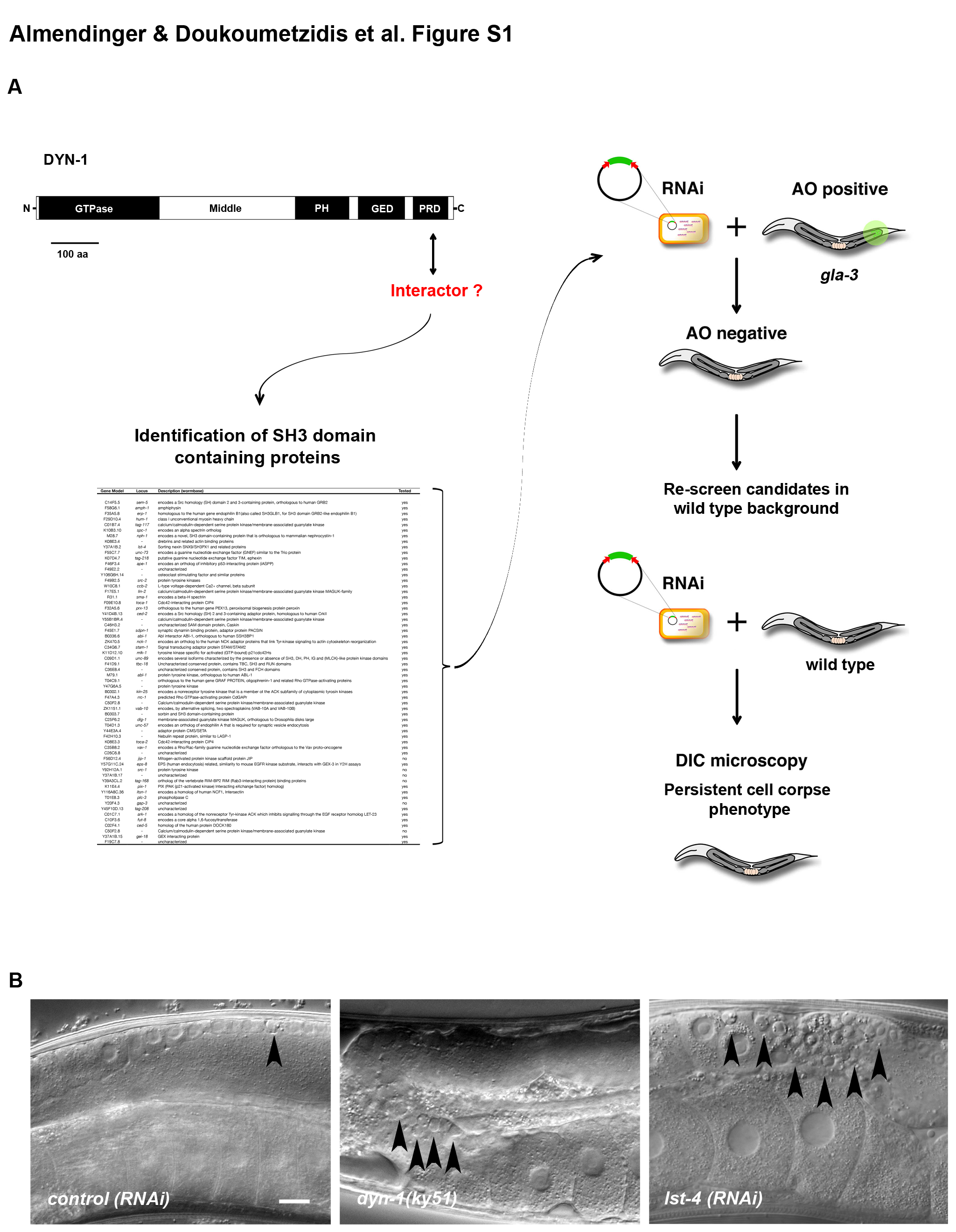

Supplement: Figure S1 — Identification of LST-4 from a targeted RNAi screen in C. elegans . (A) Schematic of the candidate based reverse genetic screen. The C. elegans DYN-1 contains beside other domains, a C-terminal proline rich domain (PRD) [1]. PRDs are known to bind to SH3 domains of effector proteins [2]. We thus set up a list of all C. elegans genes encoding for SH3 domain-containing proteins in order to identify effector proteins that might function together with DYN-1 during cell corpse clearance. As previously described dyn-1 (RNAi) treated or dyn-1(ky51) mutant worms at the non-permissive temperature accumulate engulfed apoptotic germ cell corpses within non-acidified (Acridine Orange negative) phagosomes [3]. In a first round of screening we thus fed bacteria expressing dsRNA using the Ahringer RNAi feeding library [4] for our candidate genes to gla-3(op216) mutant worms at the L1 larval stage, then adults were stained with acridine orange (AO). gla-3(op216) mutant worms were used to increase the number of apoptotic cell corpses to allow the observation of acridine orange staining under a dissecting microscope. Candidates, which were able to suppress AO staining, underwent a second round of screening in N2 wild type animals and analyzed for increased germ cell corpses in the adult hermaphrodite germ line. (B) DIC micrographs of C. elegans adult hermaphrodites germ lines at the stage 24 h post L4/adult molt. RNAi was performed on N2 wild type animals as previously described. Compared to control RNAi, RNAi-mediated knockdown of lst-4 resulted in persistent cell corpses, a phenotype which is similar to dyn-1(ky51) mutants at the non-permissive temperature. Interestingly in lst-4(RNAi) or lst-4(tm2423) animals we were unable to observe any obvious defect in the clearance of embryonic cell corpses. It might be that lst-4 is not expressed, or perhaps simply redundant under these conditions. The latter hypothesis is supported by recent work from Yang and coworkers, who found that the [file pone.0018325.s001.tif]

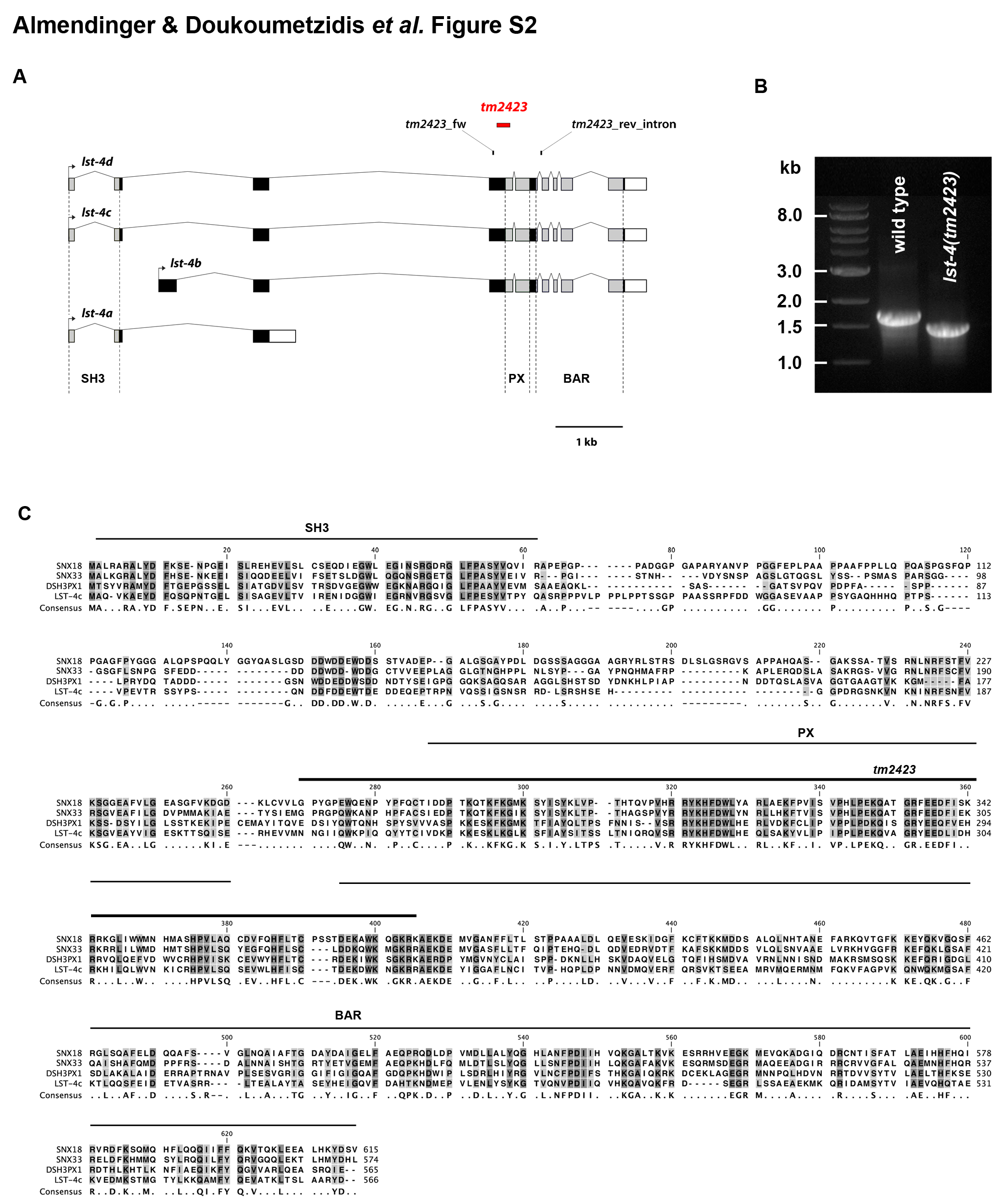

Supplement: Figure S2 — Alignment and molecular nature of lst-4 and its allele tm2423 . (A) The C. elegans lst-4 locus is predicted to code for at least four different isoforms: lst-4a, lst-4b, lst-4c and lst-4d. We confirmed by RT-PCR amplification and subsequent sequencing the exon/intron structure for the two isoforms lst-4b and lst-4c. Boxes represent exons; the regions coding for the SH3, the PX or the BAR domain are highlighted in grey, white boxes represent 3′ untranslated regions. Thin lines represent introns. tm2423 is a 212 bp deletion that results in a frame shift and premature termination (red bar). The positions of the primers used for genotyping tm2423 are indicated. (B) Genotyping of lst-4(tm2423) and wild type animals by PCR amplification. Primer sequences are described in Table S2. (C) Protein sequence alignment of C. elegans LST-4c with human SNX18, mouse SNX33, and Drosophila DSH3PX1. All proteins contain a similar protein architecture consisting of a conserved N-terminal SH3 domain, a middle PX domain and a C-terminal Bar domain (indicated by thin lines). The thick line indicates the location of the tm2423 deletion. The tm2423 deletion results in truncated protein lacking the PX domain and the whole C-terminal part (Fig. 1I). (TIF) [file pone.0018325.s002.tif]

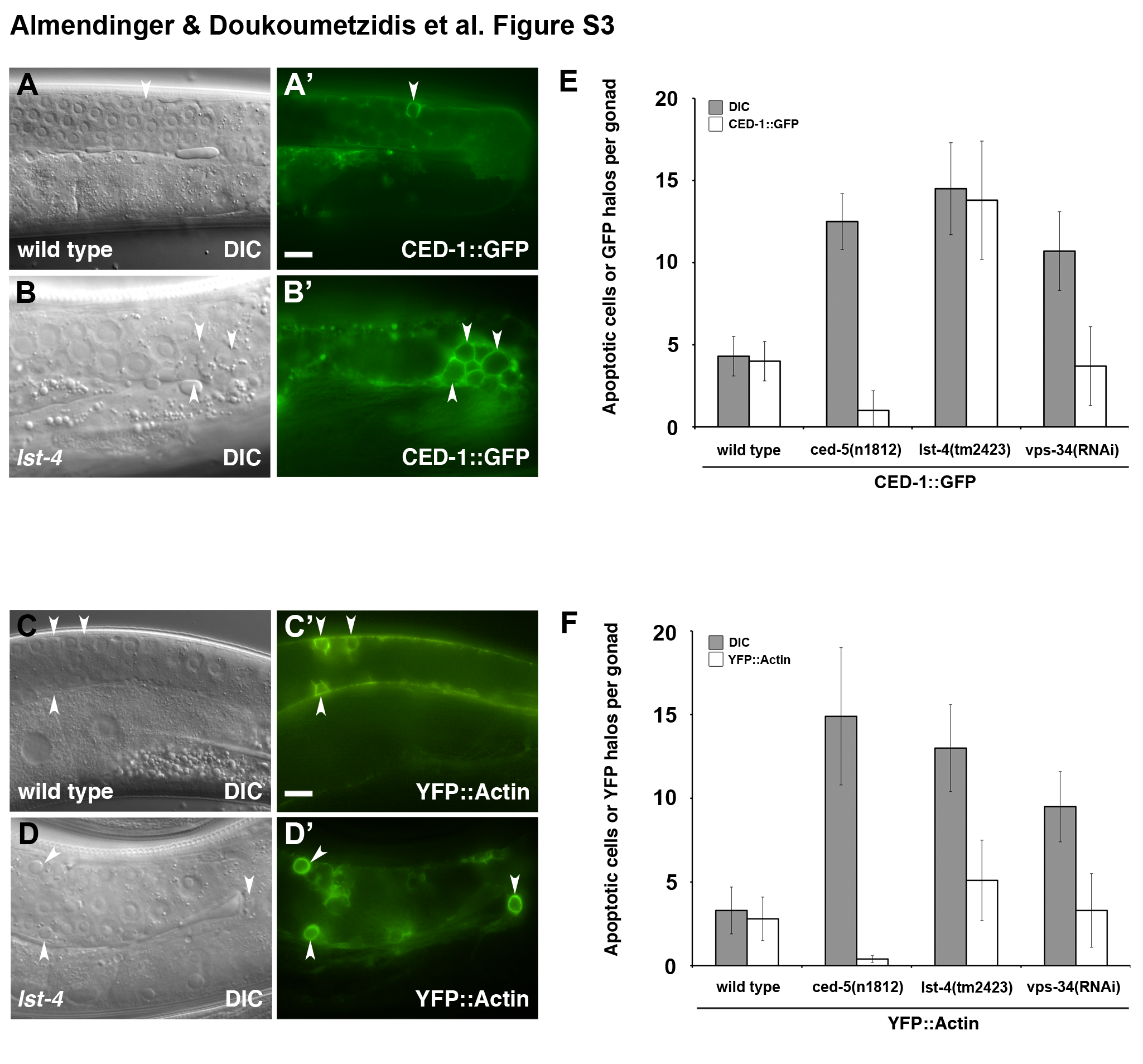

Supplement: Figure S3 — Corpses are efficiently recognized and internalized in lst-4(tm2423) mutant worms. (A–D) DIC (A′–D′) and epifluorescence images of CED-1::GFP (A′, B′) and YFP::actin (C′, D′) in wild-type (A, C) and lst-4 mutants (B, D). Arrowheads indicate apoptotic germ cells or protein around apoptotic germ cell. In lst-4(tm2423) mutants, the recruitment of CED-1::GFP (B′) and the reorganization of YFP::actin (D′) during engulfment appear normal. Size bar, 10 mm. (E, F) Quantification of germ cell corpses and CED-1::GFP (E) or YFP::actin halos (F) around apoptotic cells in the indicated genetic backgrounds. Animals were scored 24 h post L4/adult molt under DIC and epifluorescence. Data shown are means ± SD, n>15 animals. (TIF) [file pone.0018325.s003.tif]

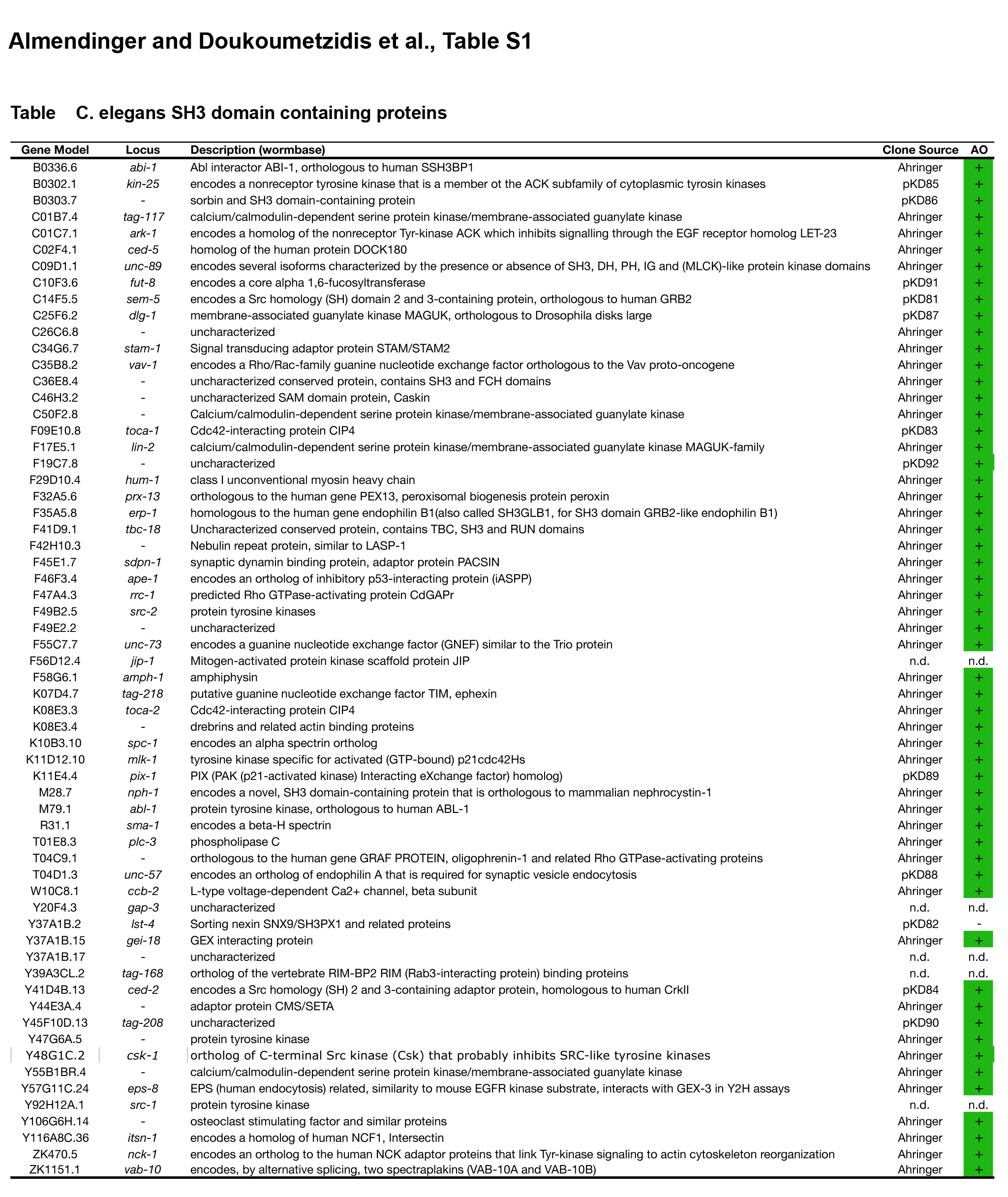

Supplement: Table S1 — List of C. elegans genes, encoding SH3-containing proteins, screened for suppression of AO staining. RNAi was performed in the gla-3(op216) background (to asses suppression of AO) or in wild-type worms where applicable (to quantify persistent cell corpses) as described in materials and methods. Only RNAi against lst-4 was found to potently suppress AO staining of apoptotic germ cell corpses and to provoke a strong cell corpse accumulation in the C. elegans germline. The known SH3 domain containing engulfment genes ced-2 and ced-5 were not identified in this screen, likely due to the variable penetrance of feeding RNAi against these two genes. Clone source: Ahringer: plasmids from the Ahringer RNAi library [6]. pKD clones: genomic fragments from the gene of interest were PCR amplified and cloned into the RNAi feeding vector L4440. n.d., not done. (For Supplemental References see File S1). (TIF) [file pone.0018325.s004.tif]

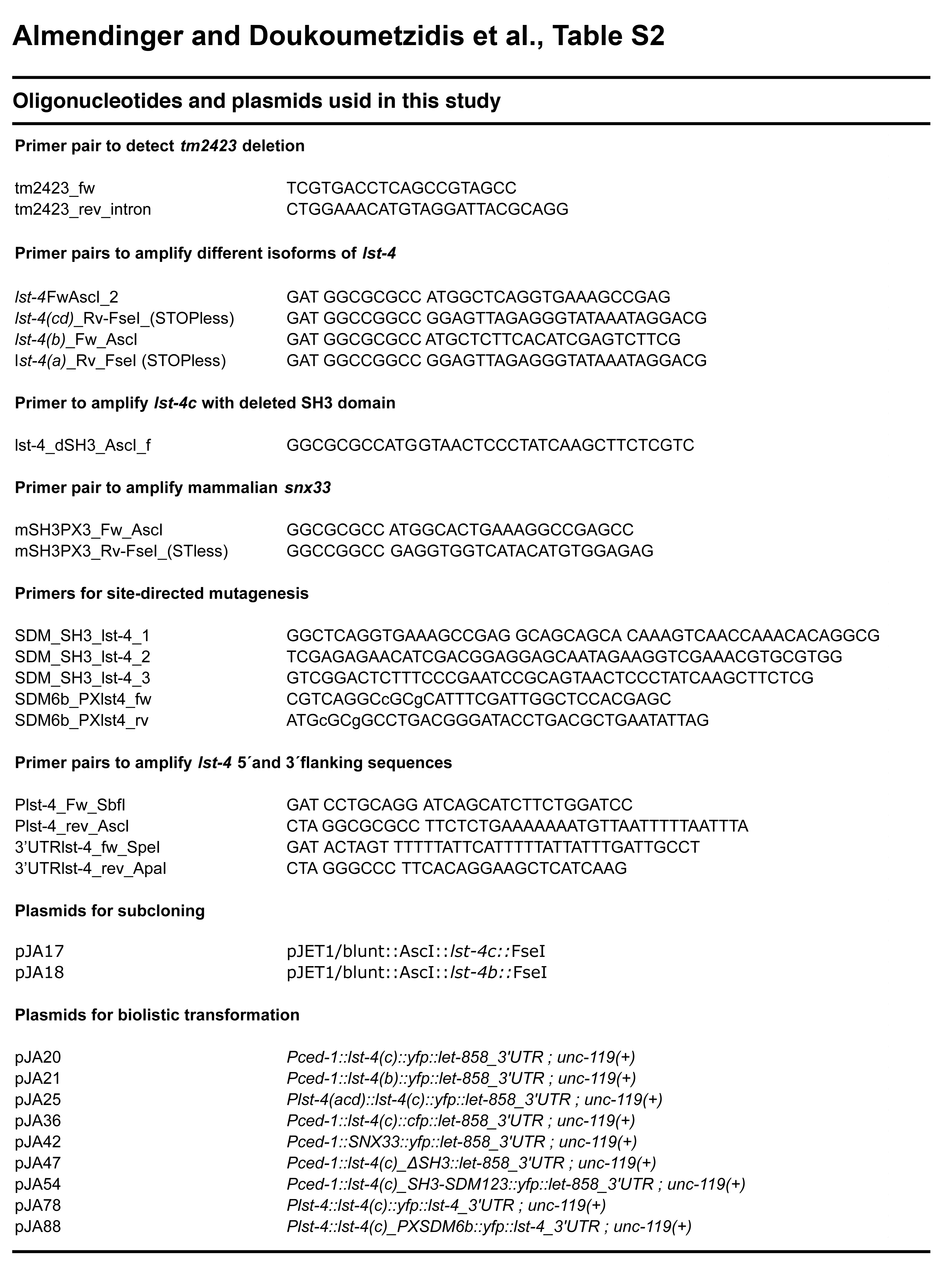

Supplement: Table S2 — List of Primers and Plasmids used in this study. (TIF) [file pone.0018325.s005.tif]
