# Supplementary material for: A Conserved Role for SNX9-Family Members in the Regulation of Phagosome Maturation during Engulfment of Apoptotic Cells
Source: PLoS One. 2011 Apr 8;6(4):e18325. doi: 10.1371/journal.pone.0018325 (PMC3072968; doi:10.1371/journal.pone.0018325)
Supplement: File S1 — Supplemental References. (DOCX) [file pone.0018325.s006.docx]

**Supplemental References**

1. Clark SG, Shurland DL, Meyerowitz EM, Bargmann CI, van der Bliek AM (1997) A dynamin GTPase mutation causes a rapid and reversible temperature-inducible locomotion defect in C. elegans. Proc. Natl. Acad. Sci. U.S.A 94: 10438-10443.

2. Gout I, Dhand R, Hiles ID, Fry MJ, Panayotou G, et al. (1993) The GTPase dynamin binds to and is activated by a subset of SH3 domains. Cell 75: 25-36.

3. Kinchen JM, Doukoumetzidis K, Almendinger J, Stergiou L, Tosello-Trampont A, et al. (2008) A pathway for phagosome maturation during engulfment of apoptotic cells. Nat. Cell Biol 10: 556-566.

4. Kamath RS, Ahringer J (2003) Genome-wide RNAi screening in Caenorhabditis elegans. Methods 30: 313-321.

5. Chen D, Xiao H, Zhang K, Wang B, Gao Z, et al. (2010) Retromer is required for apoptotic cell clearance by phagocytic receptor recycling. Science 327: 1261-1264.

6. Kamath RS, Fraser AG, Dong Y, Poulin G, Durbin R, et al. (2003) Systematic functional analysis of the Caenorhabditis elegans genome using RNAi. Nature 421: 231-237.
